# Supplementary figures and images for: Identification of a Green Algal Strain Collected from the Sarno River Mouth (Gulf of Naples, Italy) and Its Exploitation for Heavy Metal Remediation
Source: Microorganisms. 2022 Dec 10;10(12):2445. doi: 10.3390/microorganisms10122445 (PMC9781626; doi:10.3390/microorganisms10122445)

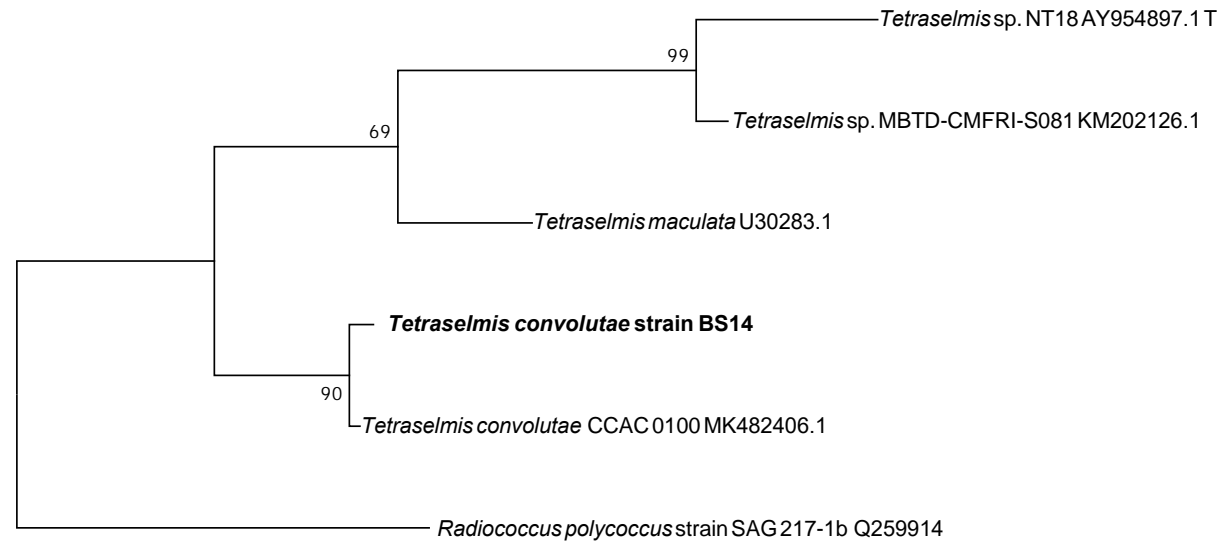

0.020

Supplement: Supplementary file 1 [file microorganisms-10-02445-s001.zip › microorganisms-2082475-supplementary.pdf]
